# A radiomics‐based nomogram may be useful for predicting telomerase reverse transcriptase promoter mutation status in adult glioblastoma

**DOI:** 10.1002/brb3.3528

**Published:** 2024-05-26

**Authors:** Yao Li, Ling Chen, Lizhao Huang, Xuedong Li, Qidan Huang, Lifang Tang, Zhiwei Huang, Li Zhu, Tao Li

**Affiliations:** ^1^ Department of Neurosurgery Liuzhou Worker's Hospital Guangxi China; ^2^ Department of Radiology Liuzhou Worker's Hospital Guangxi China

**Keywords:** glioblastoma, magnetic resonance imaging, nomogram, radiomics, telomerase reverse transcriptase

## Abstract

**Background and purpose:**

As a crucial diagnostic and prognostic biomarker, telomerase reverse transcriptase (TERT) promoter mutation holds immense significance for personalized treatment of patients with glioblastoma (GBM). In this study, we developed a radiomics nomogram to determine the TERT promoter mutation status and assessed its prognostic efficacy in GBM patients.

**Methods:**

The study retrospectively included 145 GBM patients. A comprehensive set of 3736 radiomics features was extracted from preoperative magnetic resonance imaging, including T2‐weighted imaging, T1‐weighted imaging (T1WI), contrast‐enhanced T1WI, and fluid‐attenuated inversion recovery. The construction of the radiomics model was based on integrating the radiomics signature (rad‐score)with clinical features. Receiver‐operating characteristic curve analysis was employed to evaluate the discriminative ability of the prediction model, and the risk score was used to stratify patient outcomes.

**Results:**

The least absolute shrinkage and selection operator classifier identified 10 robust features for constructing the prediction model, and the radiomics nomogram exhibited excellent performance in predicting TERT promoter mutation status, with area under the curve values of.906 (95% confidence interval [CI]:.850–.963) and.899 (95% CI:.708–.966) in the training and validation sets, respectively. The clinical utility of the radiomics nomogram is further supported by calibration curve and decision curve analyses. Additionally, the radiomics nomogram effectively stratified GBM patients with significantly different prognoses (HR = 1.767, *p *= .019).

**Conclusion:**

The radiomics nomogram holds promise as a modality for evaluating TERT promoter mutations and prognostic outcomes in patients with GBM.

## Graphical abstract

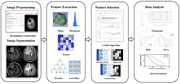

## 1 INTRODUCTION

Glioblastoma (GBM) is the most common and aggressive primary malignant brain tumor in adults, accounting for approximately 30% of all brain tumors (Chen et al., 2022; Omuro & DeAngelis, 2013). Despite advances in treatment modalities such as surgery, radiation therapy, and chemotherapy, the prognosis for GBM remains poor, with a median survival of only 15 months (Nam & De Groot, 2017; Wick et al., 2018). The high recurrence rate and therapy resistance contribute to the unfavorable outcomes observed in patients with GBM. However, recent developments have shed light on potential genetic alterations that may aid in the precise categorization and characterization of these tumors. In 2021, the incorporation of telomerase reverse transcriptase (TERT) promoter genetic alterations into the diagnostic classification of GBM within the central nervous system brain tumor taxonomy has provided novel genetic insights that facilitate precise categorization and characterization of these tumors (Gritsch et al., 2022; Kurokawa et al., 2022). Furthermore, studies have shown that TERT promoter mutations have been found to be associated with a more aggressive form and a worse prognosis (Ivanidze et al., 2019; Li et al., 2020). This discovery opens up new possibilities for targeted therapeutic interventions aimed at addressing this specific mutation.

In the majority of somatic cells, the TERT gene remains inactive, meaning it is not expressed or functioning. However, in approximately 80% of cases of GBM, there is a reactivation of the TERT gene. This reactivation occurs due to high‐frequency mutations that take place in the promoter region of the gene. The presence of TERT promoter mutations has been associated with increased TERT expression and telomerase activity (Kent et al., 2019; Yik et al., 2021). TERT is the catalytic subunit of the telomerase enzyme that adds telomeric repeats to the ends of chromosomes, preventing their degradation and ensuring the stability of the genome (He et al., 2022; Mancini et al., 2018). Mutations in the TERT promoter region result in upregulation of gene expression, thereby inducing the activation of telomerase activity and conferring tumor cells with unlimited proliferative potential (Heidenreich et al., 2015; Tahara et al., 1999; Patel et al., 2020). Recent studies have demonstrated that GBM with TERT promoter mutation and MGMT promoter unmethylation is associated with decrease sensitivity to radiotherapy and temozolomide (TMZ) treatment (Vuong et al., 2020). The TERT promoter mutation leads to increased telomerase activity, allowing cancer cells to maintain their telomeres and avoid senescence or cell death (Lorbeer & Hockemeyer, 2020). Consequently, GBM tumors harboring this mutation exhibit enhanced resistance to radiation therapy as well as TMZ chemotherapy.

DNA sequencing is widely recognized as the gold standard technique for detecting TERT mutations, particularly in the context of diagnosing and treating GBM. However, it is important to acknowledge that this approach may have certain limitations when applied in a clinical setting. One potential limitation of relying solely on DNA sequencing is that it requires obtaining tissue samples through invasive procedures such as biopsies or surgeries. For some GBM patients who cannot undergo surgery due to various reasons like advanced age or poor overall health conditions, this poses a significant challenge. Furthermore, the presence of tumor heterogeneity adds another layer of complexity. GBM tumors are known for their intratumoral heterogeneity, meaning that different regions within a single tumor can exhibit distinct genetic profiles. This poses difficulties when obtaining representative samples for DNA sequencing analysis since a small biopsy might not fully capture all relevant mutations present throughout the tumor mass. In such cases, alternative diagnostic techniques that offer enhanced accessibility and noninvasiveness can be considered from the initial stages of tumor diagnosis in GBM patients.

Radiomics, as an emerging technology in the field of medical imaging, holds great promise for providing valuable insights into gliomas. By analyzing various quantitative image features extracted from magnetic resonance imaging (MRI) scans using radiomics techniques, researchers have been able to identify potential biomarkers associated with TERT promoter mutations in gliomas. These biomarkers can provide important information about the genetic characteristics and aggressiveness of these tumors. Previous reports have demonstrated that specific radiomic features, including tumor shape, texture, and enhancement patterns, can provide valuable insights for predicting TERT promoter mutations and survival outcomes (Fang et al., 2020; Ivanidze et al., 2019). More recently, several researchers have employed multi‐parameter MRI radiomics analysis to effectively differentiate TERT mutant gliomas from wild‐type counterparts with promising results (He et al., 2022; Park, Han et al., 2021; Tian et al., 2020; Wang et al., 2023). However, these studies have predominantly focused on grade 2–4 gliomas, and there is a limited amount of literature regarding the utilization of radiomics analysis to differentiate TERT promoter status in patients with GBM. Consequently, this study aims to establish a radiomics model capable of predicting both TERT promoter mutation status and prognosis in GBM patients.

## 2 MATERIALS AND METHODS

### 2.1 Patients

**FIGURE 1:**
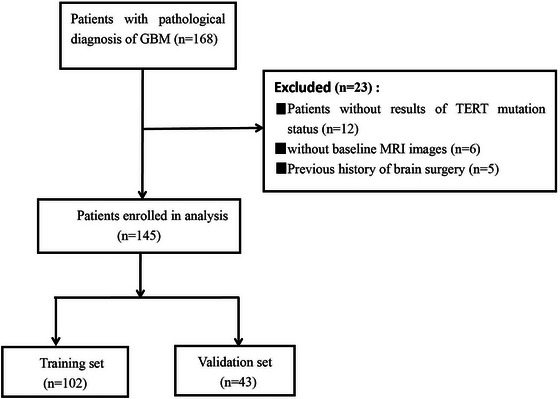
Inclusion of patient flowchart.

The retrospective study was approved by the institutional research ethics review board, and patient consent requirements were waived. A total of 168 GBM patients were enrolled in the study between January 2018 and January 2023. All patients underwent surgical resection followed by chemoradiotherapy. Clinical data were gathered from the hospital information system, including age, sex, overall survival (OS) in months, preoperative Karnofsky performance status (KPS), tumor size, and genetic information (with or without TERT promoter). The diagnosis of GBM patients includes diffuse astrocytoma with wild‐type IDH expression followed by one or more histological or genetic changes: endothelial cell proliferation, necrosis, TERT promoter mutation, EGFR gene amplification, and +7/−10 chromosome copy number variation. The MRI images were obtained from the picture archiving and communication systems (PACS). The inclusion criteria were as follows: (1) Individuals aged over 18 years; (2) no history of brain surgery or chemoradiotherapy; (3) availability of preoperative MRI imaging data; (4) pathological diagnosis consistent with the study. On the other hand, a total of 23 patients were excluded due to (1) patients without available TERT mutation status results, (2) absence of baseline MRI data, and (3) diagnosis of other types of brain tumors. The patient selection flowchart is shown in Figure 1.

### 2.2 MRI protocol

MR imaging data included axial T2‐weighted imaging (T2WI), T1‐weighted imaging (T1WI), contrast‐enhanced T1WI (CE‐T1WI), and fluid‐attenuated inversion recovery (FLAIR) sequences obtained on 1.5T MRI system (GE, Octane; Siemens, Altea) and 3.0T MRI system (Philips, Achieva; GE, Premier). Post‐contrast T1WI were taken following intravenous injection of gadoterate meglumine through the median cubital vein at a flow rate of 2 mL/s (0.2 mL/kg body weight). The MRI parameters are provided in Supplementary 1.

### 2.3 Radiomics process

#### 2.3.1 Image preprocessing and segmentation

**FIGURE 2:**
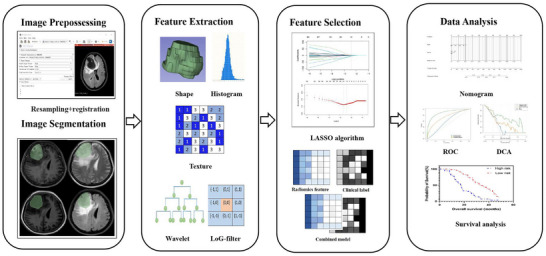
Radiomics processing flowchart.

DICOM images from T2WI, T1WI, CE‐T1WI, and FLAIR were imported into 3D Slicer software (version 5.3.0; https://www.slicer.org/). For each case, image preparation was standardized to include resampling (voxel size: 1 mm × 1 mm × 1 mm), intensity normalization (*Z*‐score), and discretization (bin width: 25). Volume of interest was delineated manually along the enhancing margin slice by slice on CE‐T1WI and registered to T1WI, T2WI, and FLAIR images. It is important to note that necrosis, calcification, bleeding, and edema lesions were not included in the analysis. Tumor segmentation was carried out by two neuroradiologists (L.Z.H and L.F.T) with 10 years of experience in neuroradiology. In addition, the accuracy of the pathological diagnosis was further ensured through a rigorous process involving two experienced neuropathologists. The inter‐class correlation value ranged between 0.85 and 1, showing a high level of consistency. Any differences between the two neuroradiologists were settled by consensus. The radiomics process is shown in Figure 2.

#### 2.3.2 Feature extraction

Radiomics feature extraction was performed using FeAture Explorer (https://github.com/salan668/ FAE, version 0.3.7) on Python (3.7.6). These features were divided into five categories, including 14 shape features, 18 first‐order features, 75 texture features: 24 gray level co‐occurrence matrix, 16 gray level run length matrix, 14 gray level dependence matrix, 16 gray level size zone matrix, 5 neighborhood gray‐tone difference matrix, 744 wavelet transform, and 93 Laplacian of Gaussian filter. Finally, a total of 3736 (934 × 4) features were extracted for each patient. Details of the feature are shown in Supplementary 2.

#### 2.3.3 Feature selection and model establishment

The datasets were randomly divided into training set (102 cases) and validation set (43 cases) in a ratio of 7:3. To identify the most pertinent and robust features for radiomics analysis, a two‐step process was employed in radiomics. Initially, the training cohort underwent either the Mann–Whitney *U*‐test or independent *t*‐test to eliminate features with a *p*‐value exceeding.05. The remaining features were considered relevant for distinguishing TERT mutation status at baseline. Subsequently, the least absolute shrinkage and selection operator (LASSO) algorithm was employed for further feature selection. A rad‐score, based on LASSO, was generated by retaining optimal features with nonzero coefficients. Finally, a radiomics nomogram integrating the rad‐score and clinical features was constructed using multivariate logistic regression.

The radiomics nomogram was verified on the validation cohort. The performance of the receiver‐operating characteristic (ROC) curve was assessed based on metrics, including area under the curve (AUC), cutoff, accuracy, sensitivity, specificity, positive predictive value, and negative predictive value. The DeLong test was used to compare the AUC of the ROC curves and determine the 95% confidence intervals (95% CIs) for AUC values. The calibration curve was employed to evaluate the degree of agreement between the predicted probability and the observed outcomes across different levels of risk. The Hosmer–Lemeshow test was conducted to evaluate the fit of all models. The decision curve analysis (DCA) was performed to quantify the net benefits under different threshold probabilities in the validation set.

#### 2.3.4 Prognosis analysis

The patients were stratified into predicted TERT mutant‐type (TERT‐mt) and predicted TERT wild‐type (TERT‐wt) subgroups based on the nomogram risk scores. Subsequently, we employed the aforementioned predictive model and the actual pathologically diagnosed TERT mutation state model for prognostic analysis. Kaplan–Meier curves were employed to assess the OS of GBM patients in relation to both risk stratification groups and the pathological diagnosis of TERT mutant status. The log‐rank test was conducted to determine any disparities in survival between these two groups.

#### 2.3.5 Statistical analysis

SPSS 27.0 (version 27.0; IBM) and R statistical software (version 4.0.2) were used for statistical analyses. The independent‐samples *t*‐test or Mann–Whitney *U*‐test was used for continuous variables. The chi‐square test or Fisher's exact test was used for categorical variables. *p *< .05 values were considered indicative of statistical significance.

## 3 RESULTS

### 3.1 Basic clinical information of the patients

The basic clinical characteristics of the GBM patients are shown in Table 1. A total of 145 patients (63 females, 82 males; mean age, 50.37 ± 14.62 years [range, 37–74]) were included in this study. Of these, 45.5% cases (66/145) had TERT mutations, whereas 54.5% (79/145) did not. There were no sex significant distribution differences among the TERT subgroups. However, the age and KPS score were significantly different in the TERT mutation subgroups (*p *< .05).

**TABLE 1:** The basic clinical characteristics of GBM patients.

Variables	Overall	Train (*N* = 102)	Test (*N* = 43)	*p*
Gender				
Female	63 (43.4%)	44 (43.1%)	19 (44.2%)	.907
Age	57.88 ± 7.76	57.71 ± 7.89	58.28 ± 7.52	.686
KPS	78.07 ± 14.92	78.73 ± 13.25	76.51 ± 18.37	.138
OS	24.66 ±9.06	24.99 ± 9.07	23.83 ± 9.07	0.489
Tumor size	103.28 ± 49.84	105.53 ± 51.32	97.93 ± 46.28	.156
TERT‐mt	66 (45.5%)	51 (50%)	15 (34.9%)	.876

Abbreviations: KPS, Karnofsky performance status; OS, overall survival; TERT‐mt, telomerase reverse transcriptase mutant‐type;

### 3.2 Radiomics feature selection and model construction

**FIGURE 3:**
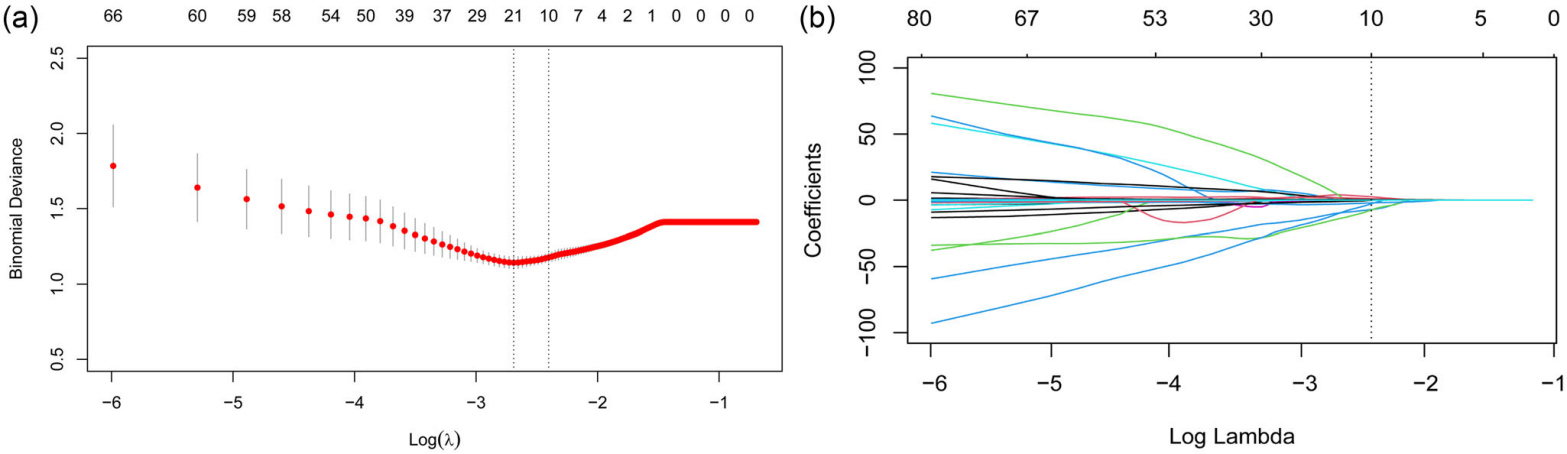
Feature selection using the least absolute shrinkage and selection operator (LASSO) method. The minimal criterion and the one standard error of the minimum criteria were represented as two vertical lines (a). Ten robust features with nonzero coefficients were finally preserved, as the vertical line was drawn (b).

Out of the 3736 extracted features, baseline analysis excluded 2800 features, and further application of the LASSO algorithm with optimal regularization weight *λ* (log*λ* = −2.463) resulted in the selection of only 10 non‐zero coefficient features (Figure 3).

The reserved features from this pipeline are listed in Supplementary 3. Among the top 10 most robust features, 5 were extracted from CE‐T1WI (log‐3D_firstorder_RootMeanSquared, waveletHHH__firstorder_InterquartileRange, log‐3D_glrlm_GrayLevelNonUniformity, wavelet‐LLL_glcm_SumEntropy, wavelet‐LLL_glszm_SmallAreaEmphasis), three from FLAIR (including original_shape_Maximum3DDiameter, wavelet‐HHL_glcm_SumSquares, and wavelet‐HLH_ngtdm_Complexity), and two from T2WI (including log‐sigma‐1‐mm‐3D‐glcm_Idn and wavelet‐HLL_glcm_DifferenceEntropy).

**FIGURE 4:**
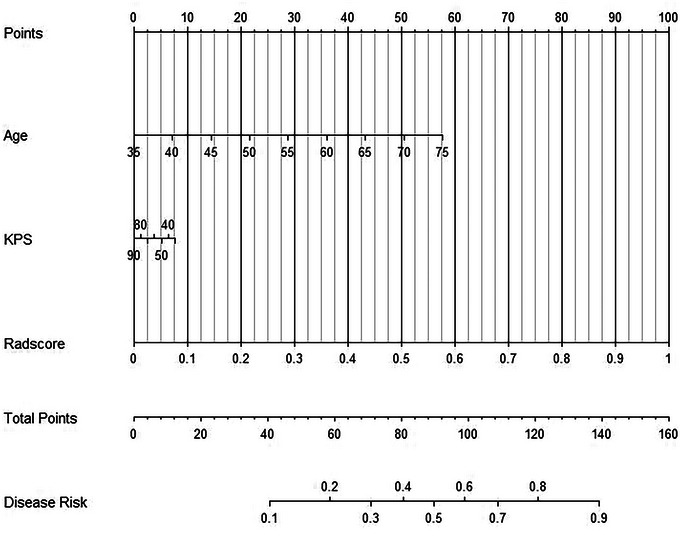
Nomogram comprised rad‐score and clinical features.

**FIGURE 5:**
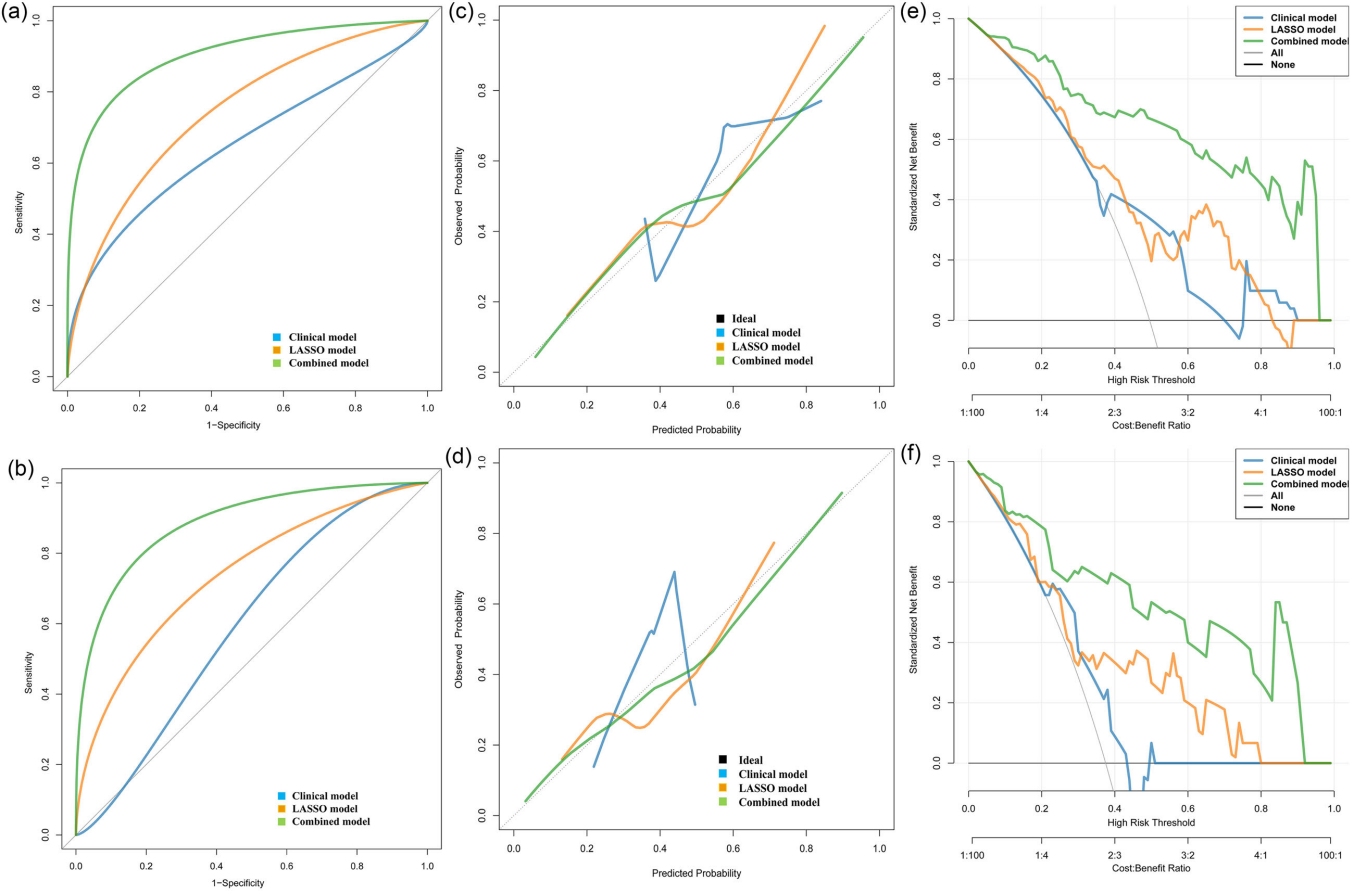
The receiver‐operating characteristic (ROC) curve results of the nomogram based on clinical features and rad‐score in the training set and validation set (a and b). Calibration curves of this nomogram for clinical model, least absolute shrinkage and selection operator (LASSO) model, and combined model in the training and validation cohorts (c and d). Analysis of clinical decision curve for the clinical model alone, LASSO model alone and clinical + LASSO combined model (e and f).

The radiomics nomogram, consisting of the rad‐score and clinical features, is presented in Figure 4. The AUC curve analyses for the clinical model, LASSO model, and combined model are depicted in Figure 5. The results demonstrated that the combined model exhibited superior diagnostic efficiency in both the training and validation sets compared to the single model (Figure 5a,b). The cutoff value, accuracy, sensitivity, specificity, and AUC in the training set were.452,.854,.843,.865, and.906, respectively. In the validation set, these values were found to be.452 for cutoff value, with an accuracy of.700, a sensitivity of.733, a specificity of.680, and an AUC of.899 (Table 2). The DeLong test revealed that the AUC values in the combined model were significantly higher than those in both the clinical model and LASSO model, both in the training set and validation set (*p* < .05). The nomogram calibration curve demonstrated a strong concordance between the predicted and observed values (Figure 5c,d). The C‐index was 0.905 in the training set and 0.834 in the validation set. The Hosmer–Lemeshow test demonstrated that the nomogram exhibited a good fit in both the training and validation sets (*p* = .567 and.895, respectively). The DCA demonstrated that the combined model outperformed both the clinical and LASSO models in predicting TERT mutation status, as depicted in Figure 5e,f.

**TABLE 2:** The area under the curve (AUC), 95% confidence interval (CI), cutoff, accuracy, sensitivity, specificity, positive predictive value (PPV), and negative predictive value (NPV) for clinical model, least absolute shrinkage and selection operator (LASSO) model, and combined model in the training set and validation set.

Models	AUC	95% CI	Cutoff	Acc	Sen	Spe	PPV	NPV	Task
**Clinical model**	.654	[.545–.764]	.550	.680	.569	.788	.725	.651	Training
	.604	[.419–.789]	.550	.575	.933	.360	.467	.900	Test
**LASSO model**	.739	[.644–.835]	.631	.728	.529	.923	.871	.667	Training
	.744	[.581–.907]	.631	.750	.600	.840	.692	.778	‐Test
**Combined model**	.906	[.850–.963]	.452	.854	.843	.865	.860	.849	Training
	.899	[.708–.966]	.452	.700	.733	.680	.579	.810	Test

### 3.3 Prognostic performance of the combined nomograms

**FIGURE 6:**
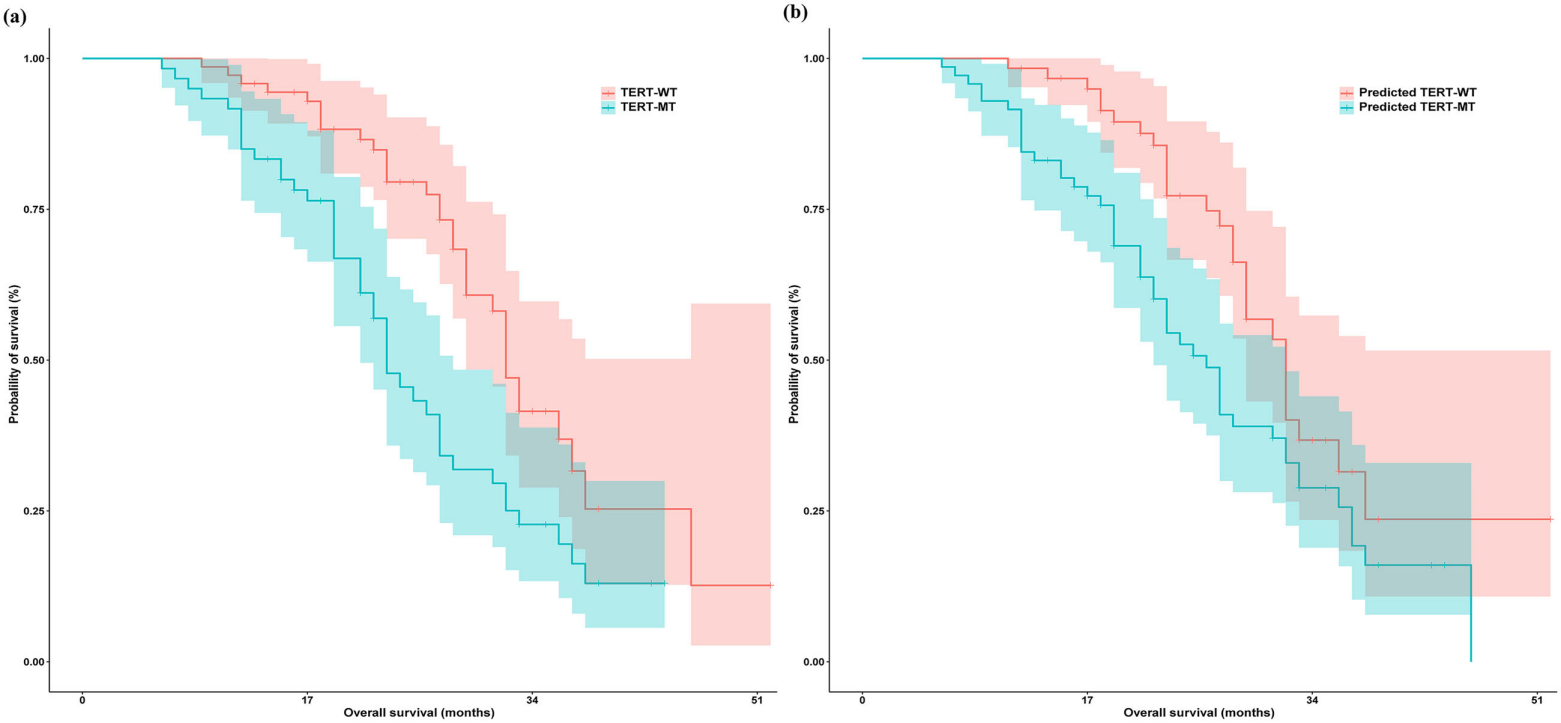
The prognostic analysis comparing the actual telomerase reverse transcriptase (TERT) promoter mutation status determined through pathological diagnosis (a) with the predicted TERT mutation status obtained from model diagnosis (b).

The prognostic study ultimately enrolled 132 patients who either survived until the expiration date or had a specific time of death, with a median follow‐up duration of 32.7 months. Through multivariate COX regression analysis, age, KPS score, and rad‐score were identified as independent prognostic factors for distinguishing TERT promoters in GBM patients (Table 3). Therefore, we utilized these factors to construct a nomogram and subsequently validated its efficacy in prognostic assessment among patients diagnosed with GBM. The prognostic analysis comparing the TERT promoter mutation status determined through pathological diagnosis with the predicted TERT mutation status obtained from model diagnosis for GBM patients is presented in Table 4 and Figure 6. According to the radiomics nomogram, patients were stratified into predicted TERT‐mt subgroups if their rad‐score exceeded 123.983, whereas those with a risk score below this threshold were classified as predicted TERT‐wt subgroups. The Kaplan–Meier curve results demonstrated a shorter OS time in the TERT‐mt subgroup compared to the TERT‐wt subgroup. This observation aligns perfectly with the predictions made by our model, which anticipated that individuals in the predicted TERT‐wt subgroup would live longer than those in the predicted TERT‐mt subgroup.

**TABLE 3:** Univariate and multivariate analyses of telomerase reverse transcriptase (TERT) promoter mutations.

Variables	Univariate		Multivariate	
	OR (95%CI)	*p*	OR (95%CI)	*p*
Age	1.050 (1.005, 1.098)	.030	1.055(.995, 1.119)	.073
KPS	.966 (.943,.989)	.005	.983(.952, 1.015)	.301
Size	.996 (.990, 1.003)	.302	/	/
Gender	1.355 (.699, 2.629)	.368	/	/
Rad‐score	128.755 (32.685, 507.200)	<.001	123.694 (30.275, 505.382)	<.001

Abbreviations: CI, confidence interval; KPS, Karnofsky performance status.

**TABLE 4:** The Kaplan–Meier curve results of the pathological diagnosis of telomerase reverse transcriptase (TERT) promoter mutation status and the predicted TERT mutation status based on radiomics signature.

Variables	coef	SE (coef)	*Z* value	HR (95%CI)	*p* Value
Predicted TERT‐wt	0.569	.242	2.348	1.767 (1.099, 2.842)	.019
Predicted TERT‐mt					
TERT‐wt	0.752	.238	3.159	2.120 (1.330, 3.380)	.002
TERT‐mt					

Abbreviations: CI, confidence interval; HR, hazard ratio; TERT‐mt, telomerase reverse transcriptase mutant‐type; TERT‐wt, telomerase reverse transcriptase wild‐type.

## 4 DISCUSSION

In this study, we conducted an investigation to explore the potential value of multiparameter MRI features in examining the association between TERT gene mutations and prognosis in GBM patients using radiomics analysis. The key findings were as follows: (1) The radiomics nomogram, consisting of the rad‐score and clinical features, demonstrated superior diagnostic efficacy compared to a single model for distinguishing TERT promoter mutation status, with AUC values of.906 and.899 in the training and validation sets, respectively. (2) Survival analysis revealed a shorter OS time in the TERT‐mt subgroup compared to the TERT‐wt subgroup. This observation aligns perfectly with the predictions made by our model, which anticipated that individuals in the predicted TERT‐wt subgroup would live longer than those in the predicted TERT‐mt subgroup. (3) Ten robust features were ultimately selected, with texture features contributing predominantly to the prediction model.

TERT gene mutations have been identified in a substantial proportion of GBM cases, with a frequency reaching up to 80% (Aquilanti et al., 2021; Giunco et al., 2023; Mosrati et al., 2015; Simon et al., 2015). These mutations augment telomerase activity, thereby facilitating the immortalization of tumor cells and expediting tumor progression (Eckel‐Passow et al., 2015; Park et al., 2021; Powter et al., 2021). In the context of gliomas, radiomics studies utilizing multiparameter MRI have demonstrated promising outcomes in predicting TERT promoter mutation status. Wang et al. (2023) employed a preoperative multiparameter MRI radiomics model to accurately discern IDH‐mutated TERT promoter mutant gliomas. The validation set yielded impressive results with an AUC of.971 and high sensitivity (.833) and specificity (.966). Calabrese et al. (2022) conducted an investigation employing an innovative artificial intelligence approach for predicting clinically relevant biomarkers in GBM. Their findings demonstrated that the combined model, incorporating radiomics and convolutional neural network, exhibited excellent performance in evaluating IDH and TERT mutation status, with an AUC exceeding.85. In a separate study, the predictive potential of radiomics features extracted from magnetic resonance spectroscopy was examined to determine the TERT mutation status among 126 patients diagnosed with high‐grade gliomas. Notably, specific MRI features, such as Lac, Cho/Cr, and rad‐score, were found to be significantly associated with TERT mutations (Tian et al., 2020). Remarkably consistent with these findings, our radiomics model demonstrated exceptional capabilities in accurately distinguishing between TERT mutant GBM and TERT wild‐type tumors.

Several studies have investigated the association between TERT mutations and prognosis in GBM patients. Kikuchi et al. (2020) conducted an analysis on the prognostic significance of radiomics features in a cohort of 153 GBM patients. These authors observed that patients harboring TERT mutations exhibited significantly reduced OS and progression‐free survival in comparison to those lacking these mutations. Furthermore, the presence of TERT mutations in tumors may indicate an elevated risk of distant lesions and a poorer prognosis. Mosrati et al. (2015) revealed that GBM patients harboring both C228 and C250 TERT promoter mutations exhibited significantly reduced OS compared to those with wild‐type TERT genes. In another study, a cohort of 273 GBM patients with IDH wild type was recruited to investigate the prognostic implications and interplay between MGMT promoter methylation and TERT promoter status (Giunco et al., 2023). Their findings revealed that the presence of the C variant allele on the TERT promoter rs2853669 served as an independent and promising prognostic biomarker for disease progression in GBM patients. Moreover, TERT mutations were found to be indicative of tumors necessitating aggressive therapeutic interventions (Juratli et al., 2018). This finding suggests that TERT promoter mutations may serve as a prognostic biomarker for GBM patients. Our study consistently demonstrated that TERT mutations are associated with a poorer prognosis in GBM patients. The Kaplan–Meier curve results demonstrated a shorter OS time in the TERT‐mt subgroup compared to the TERT‐wt subgroup. This observation aligns with the predictions made by our model. Additionally, the nomogram demonstrated a significant ability to discriminate prognostic outcomes for patients with GBM. The findings revealed that patients with predicted TERT‐wt had significantly prolonged survival compared to those with TERT‐mt. This suggests that the presence of TERT‐wt may confer a more favorable prognosis in GBM patients.

Previous studies have consistently shown the effectiveness of the nomogram in accurately predicting glioma TERT classification (Du et al., 2022; Lu et al., 2022; Tian et al., 2020). These findings highlight the value of this robust tool in clinical practice. Building upon these previous studies, our research further validates the excellent discriminatory and calibration abilities of the nomogram in patients with GBM. The nomogram, based on the multi‐factor model, is a graphical tool that allows for the integration of multiple independent variables in a prediction model. It utilizes line segments with scales to represent the relationship between these variables and their relative importance within the model. By plotting these variables on the same plane according to their fitting function relationship, it provides a visual representation of how they interact and contribute to the overall prediction. Finally, by summing up these scores for all independent variables, we can obtain a total score, which serves as an estimate for individual outcome.

The study employed radiomics features extracted from CE‐T1WI, FLAIR, T1WI, and T2WI to construct an accurate prediction model by selecting ten robust features. Among these features, texture features were found to be the most influential in differentiating TERT mutations. Following texture features, wavelet transform features, and log filter features also contributed significantly. It is worth noting that previous research has also highlighted the importance of texture features in distinguishing TERT mutations. In fact, subsets of texture features accounted for a significant proportion of the overall feature set used in this study. This finding aligns with earlier studies conducted by Fang et al. (2020), who reported that all 12 selected features exclusively consisted of texture and wavelet transform characteristics. Interestingly, another separate study also utilized predominantly texture‐based attributes for distinguishing TERT mutations. Out of the nine selected features in their analysis, eight were identified as texture‐related properties (Jiang et al., 2020).

Some limitations of our study need to be considered. First, despite the high sensitivity and specificity of our predictive model, challenges arise in interpreting and generalizing the results due to the lack of validation from other centers and the variability of imaging data obtained from different scanning devices. Additionally, advanced techniques such as amide proton transfer (Xu et al., 2021), diffusion kurtosis imaging (Hempel et al., 2021), and arterial spin labeling (Brendle et al., 2018; Hangel et al., 2023) have demonstrated promising potential in providing valuable metabolic or functional insights into tumors, respectively. These sequences offer distinctive perspectives on tumor biology that can complement conventional anatomical imaging modalities. Further investigations should prioritize the development of standardized protocols for acquiring these sequences and exploring their impact on model performance across diverse clinical scenarios.

## 5 CONCLUSION

In conclusion, the utilization of radiomics nomogram demonstrates significant potential in predicting TERT promoter mutations and patient prognosis, providing a more comprehensive assessment tool that facilitates risk stratification and treatment planning.

## AUTHOR CONTRIBUTIONS

**Yao Li**: Writing—original draft; writing—review and editing; conceptualization; resources. **Li Zhu**: Investigation; visualization; project administration; supervision. **Lizhao Huang**: Investigation; visualization; formal analysis; software. **Xuedong Li**: Conceptualization; resources; project administration. **Qidan Huang**: Investigation; conceptualization; methodology; validation; visualization. **Lifang Tang**: Formal analysis; software; data curation; supervision; project administration. **Zhiwei Huang**: Visualization; project administration; formal analysis; conceptualization; investigation. **Ling Chen**: Funding acquisition; validation; formal analysis; writing—review and editing. **Tao Li**: Conceptualization; funding acquisition; writing—review and editing; project administration.

## CONFLICT OF INTEREST STATEMENT

The authors declare that they have no known financial conflicts of interest or personal relationships that could have appeared to influence the work reported in this article.

### PEER REVIEW

The peer review history for this article is available at https://publons.com/publon/10.1002/brb3.3528.

## Supporting information

Supporting Information

## Data Availability

The data that support the findings of this study are available from the corresponding author upon reasonable request.
