# Supplementary material for: A radiomics‐based nomogram may be useful for predicting telomerase reverse transcriptase promoter mutation status in adult glioblastoma
Source: Brain Behav. 2024 May 26;14(5):e3528. doi: 10.1002/brb3.3528 (PMC11128771; doi:10.1002/brb3.3528)
Supplement: Supplementary file 1 — Supporting Information [file BRB3-14-e3528-s001.docx]

**Supplementary 1:** Magnetic Resonance Imaging Protocol.

| **parameter** | **3.0 T Philips**  **(n=46)** | **1.5 T Siemens**  **(n=18)** | **1.5 T GE**  **(n=9)** | **3.0 T GE**  **(n=72)** |
| --- | --- | --- | --- | --- |
| **T2WI** | | | | |
| TR/TE (ms) | 3600/80 | 4950/104 | 4460/126.2 | 5844/129 |
| FOV | 512*512 | 378*448 | 512*512 | 512*512 |
| FA (^。^) | 90 | 90 | 90 | 90 |
| Matrix | 256*217 | 256*203 | 512*192 | 256*217 |
| Slice thickness/gap (mm) | 5/3 | 5/2.5 | 5/2.5 | 5/2.5 |
| **T1WI** | | | | |
| TR/TE (ms) | 1200/15 | 1000/20 | 1250/15 | 1800/25 |
| FOV | 512*512 | 378*448 | 512*512 | 512*512 |
| FA (^。^) | 90 | 90 | 90 | 90 |
| Matrix | 256*217 | 256*203 | 512*192 | 256*217 |
| Slice thickness/gap (mm) | 5/3 | 5/3 | 5/3 | 5/2.5 |
| **FLAIR** | | | | |
| TR/TE (ms) | 6000/85 | 6000/85 | 6000/85 | 6500/100 |
| FOV | 230*230 | 230*230 | 230*230 | 512*512 |
| FA (^。^) | 80 | 90 | 90 | 80 |
| Matrix | 320*217 | 256*203 | 512*192 | 320*217 |
| Slice thickness/gap (mm) | 6/0.6 | 6/3 | 6/3 | 6/0.6 |

**Supplementary 2:** The classification and parameter composition of radiomics features.

| **Feature classifier Feature parameters (n=944)** |
| --- |
| Shape feature (n=14)  Elongation, Flatness, Least axis length, Major axis length, Maximum 2D diameter column, Maximum 2D diameter row, Maximum 2D diameter Slice, Maximum 3D diameter, Mesh volume, Minor axis length, Sphericity, Surface area, Surface volume ratio, Voxel volume  Histogram feature (n=18)  P10, P90, Interquartile range, Energy, Entropy, Skewness, Kurtosis, Maximum, Minimum, Mean, Mean absolute, Deviation, Median, Total energy, Uniformity, Variance, Range, Robust mean absolute deviation, Root mean squared  Texture feature (n=75)  Grey-level co-occurrence matrix, GLCM(n=24); grey-level dependence matrix, GLDM(n=14); grey-level run length matrix, GLRLM(n=16); grey-level size zone matrix, GLSZM(n=16); neighborhood gray-tone difference matrix, NGTDM (n=5)  Wavelet transform (n=744)  Wavelet filtering produces 8 decomposition per stage. In the three dimensions, all feasible combinations of high-pass or low-pass filters (LLH, LHL, LHH, HLL, HLH, HHL, HHH, LLL)  LoG filter (n=93)  Sigma-1-mm-3D-firstorder(n=18), Sigma-1-mm-3D-glcm(n=24), Sigma-1-mm-3D-gldm(n=14), Sigma-1-mm-3D-glrlm(n=16), Sigma-1-mm-3D-glszm(n=16), Sigma-1-mm-3D-ngtdm(n=5) |

**
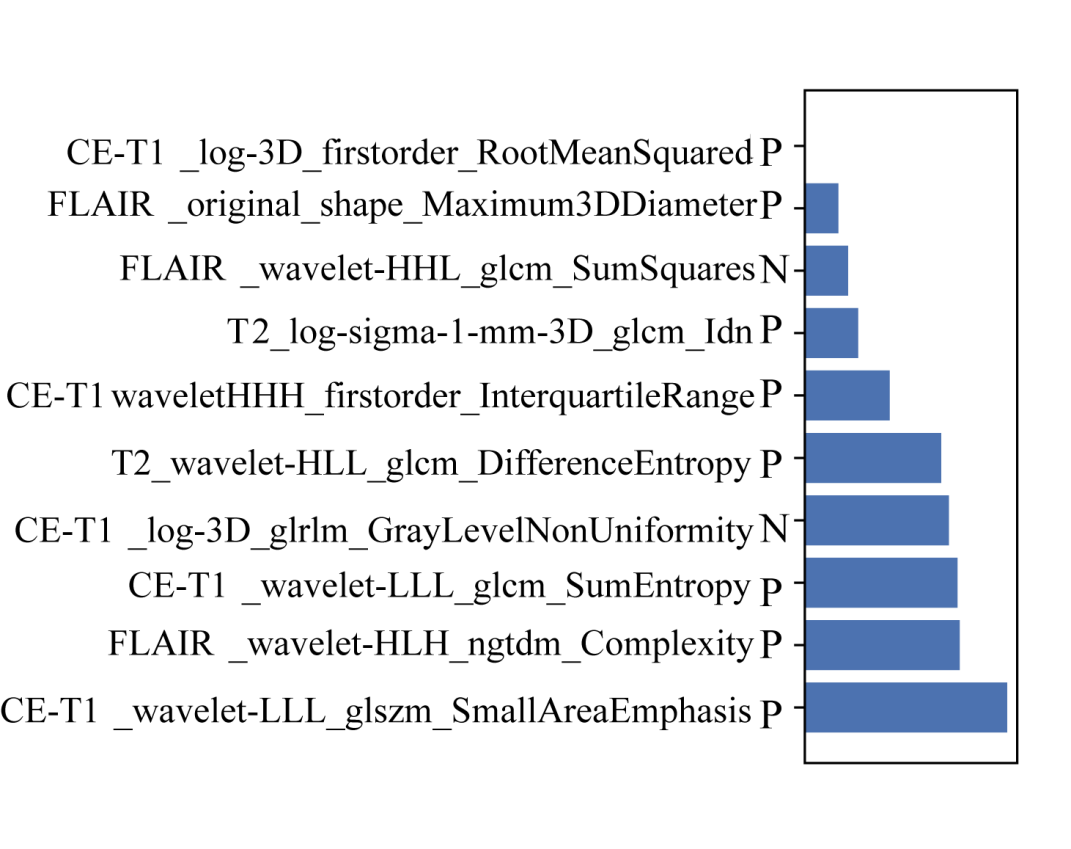
**

**Supplementary 3:** Feature weights that contribute to the LASSO model. Among the 10 most contributing features, four were extracted from CE-T1WI images, three from FLAIR images, two from T2WI images and one from T1WI images. These features are mainly characterized by the wavelet and Gaussian filter feature. Based on the nonzero coefficients of these features, the calculation formula of the radscore is as follows:

Radscore=-4.237+1.335*CE-T1_wavelet-LLL_glszm_SmallAreaEmphasis+0.4222*FLAIR_wavelet-HLH_ngtdm_Complexity+0.331*CE-T1_wavelet-LLL_glcm_SumEntropy+-0.288*CE-T1_log-3D_glrlm_GrayLevelNonUniformity+0.237*T2_wavelet-HLL_glcm_DifferenceEntropy+0.117*CE-T1_waveletHHH_firstorder_InterquartileRange+0.052*T2_log-sigma-1-mm-3D_glcm_Idn+-0.036*FLAIR_wavelet-HHL_glcm_SumSquares+0.017*FLAIR_original_shape_Maximum3DDiameter+0.002*T1_log-3D_firstorder_RootMeanSquared.
